# Supplementary material for: Anxiety and depression symptoms after COVID-19 infection: results from the COVID Symptom Study app
Source: J Neurol Neurosurg Psychiatry. 2021 Sep 28;92(12):1254–8. doi: 10.1136/jnnp-2021-327565 (PMC8599635; doi:10.1136/jnnp-2021-327565)
Supplement: Supplementary data [file jnnp-2021-327565supp001.pdf]

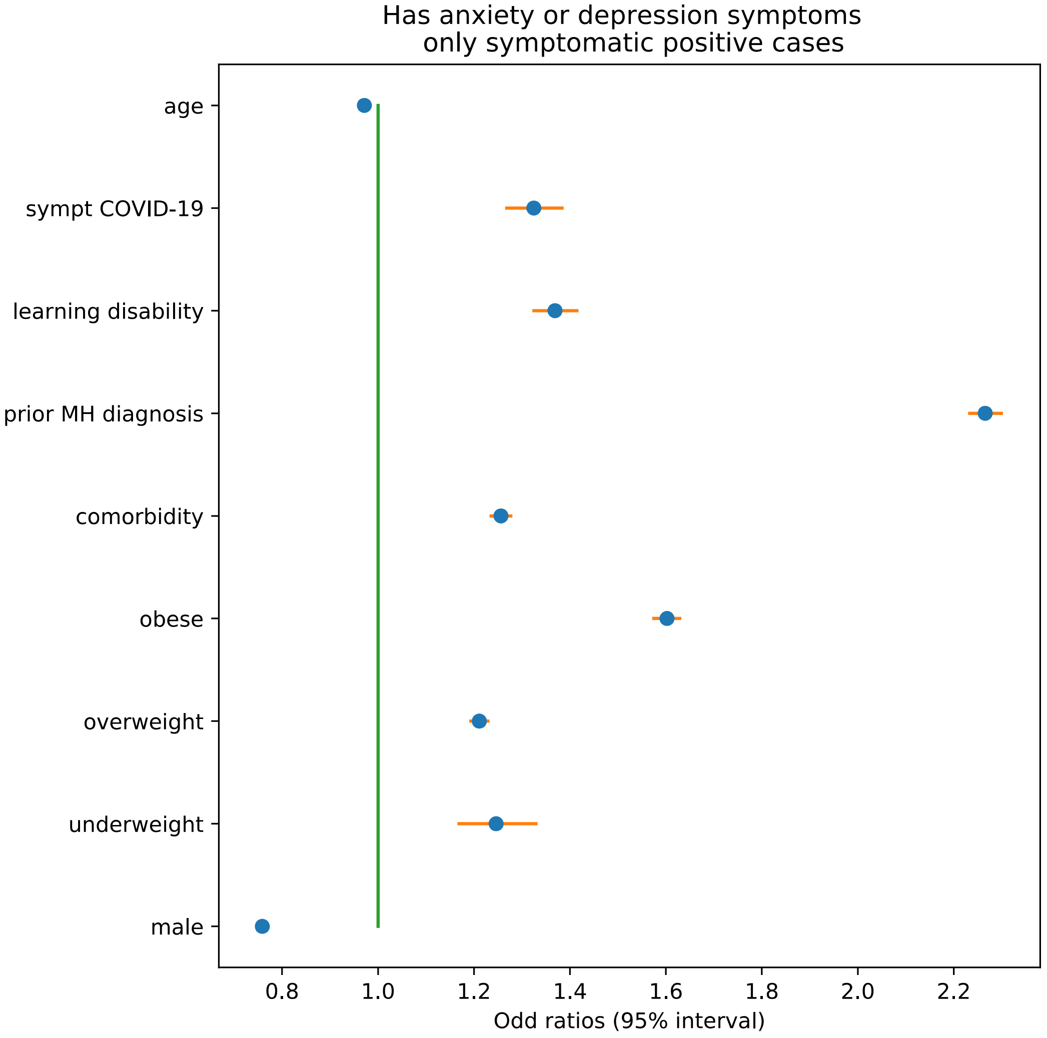

Supplementary Figure 1: Association between age, BMI, male sex, comorbidities, a previous diagnosis of a mental health (MH) condition, learning disabilities, a positive SARS-CoV-2 test result (PCR and lateral flow), and the odds ratio of anxiety/depression symptoms suggested by the results of the mental health survey. Only symptomatic cases were considered in this sensitivity analysis.

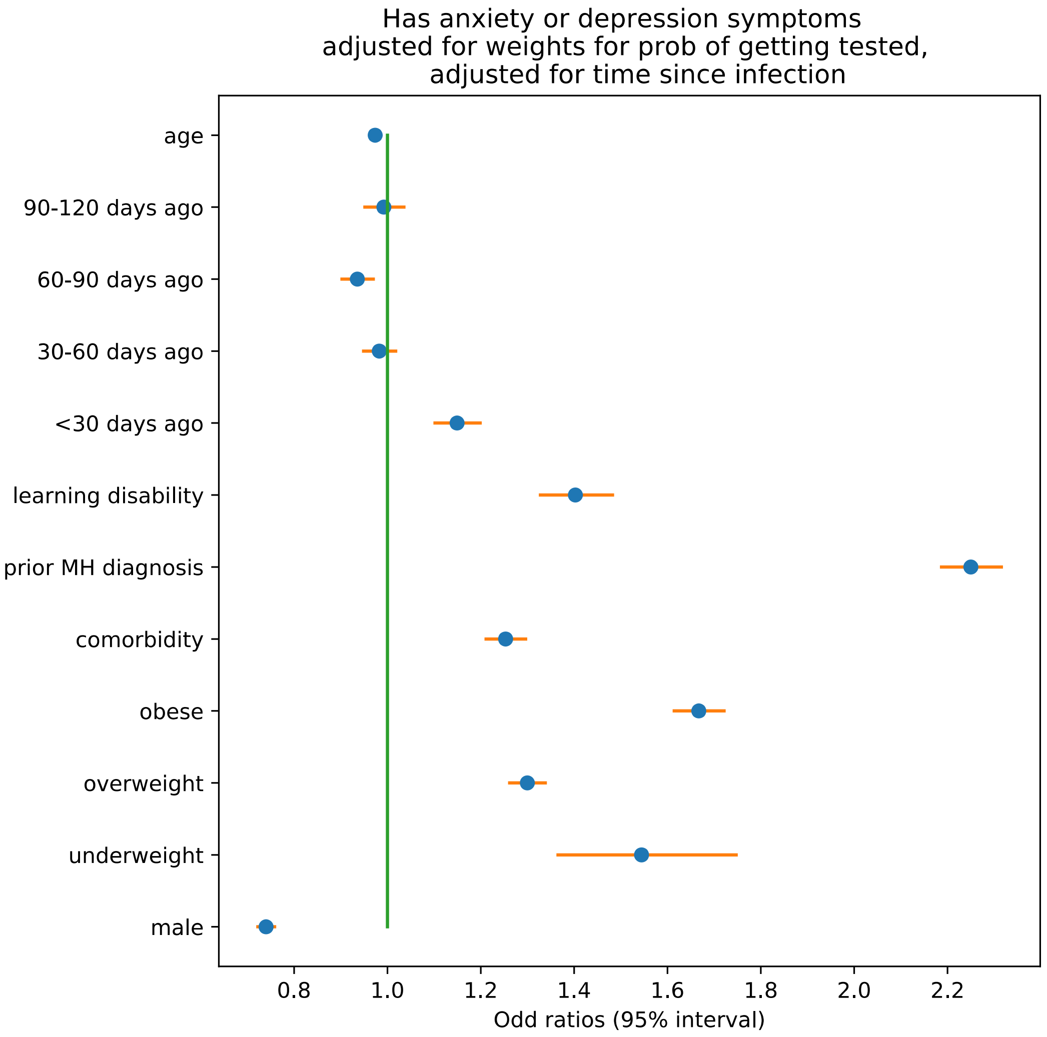

Supplementary Figure 2: Association between age, BMI, male sex, comorbidities, a previous diagnosis of a mental health (MH) condition, learning disabilities, time since infection occured, and the odds ratio of anxiety/depression symptoms suggested by the results of the mental health survey. This sensitivity analysis was performed on users with a positive SARS-CoV-2 test result (PCR and lateral flow) only.

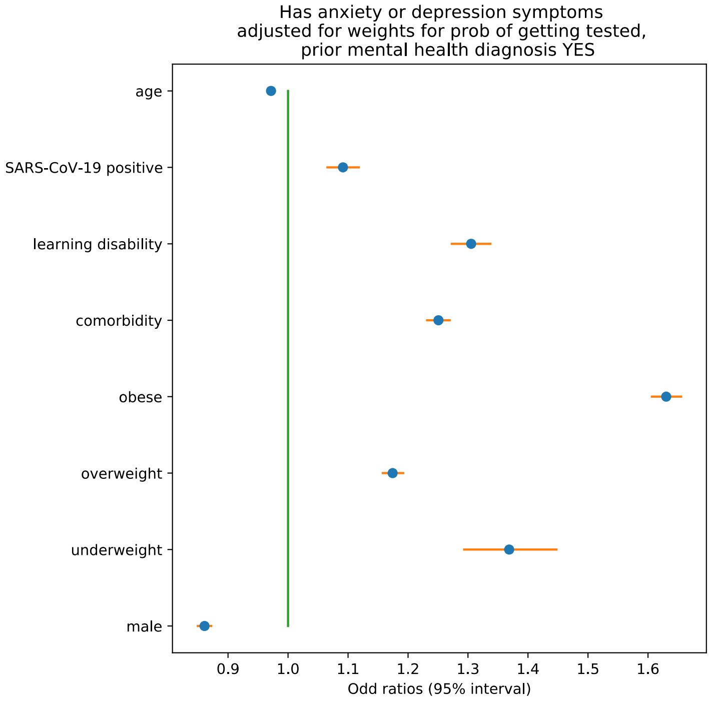

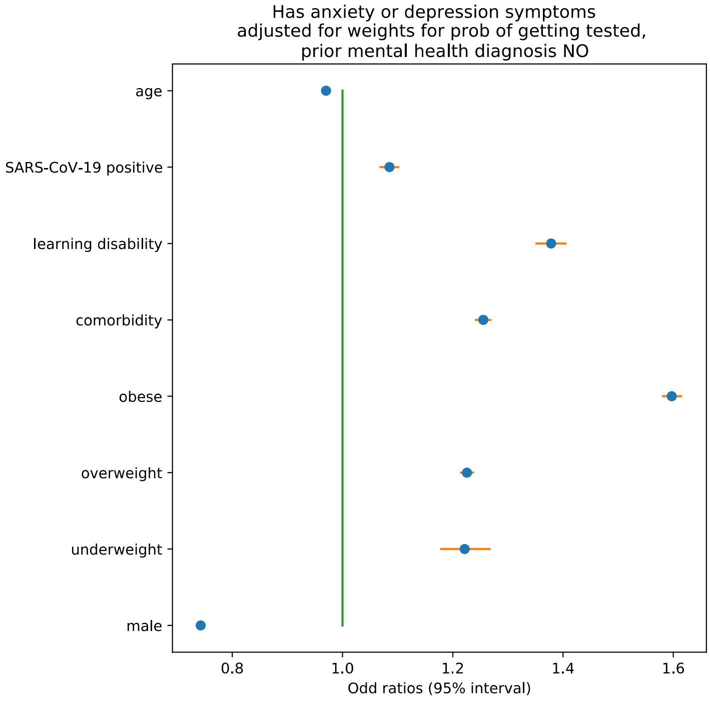

Supplementary Figure 3: Association between age, BMI, male sex, comorbidities, learning disabilities, a positive SARS-CoV-2 test result (PCR and lateral flow), and the odds ratio of anxiety/depression symptoms suggested by the results of the mental health survey stratified by prior mental health condition.

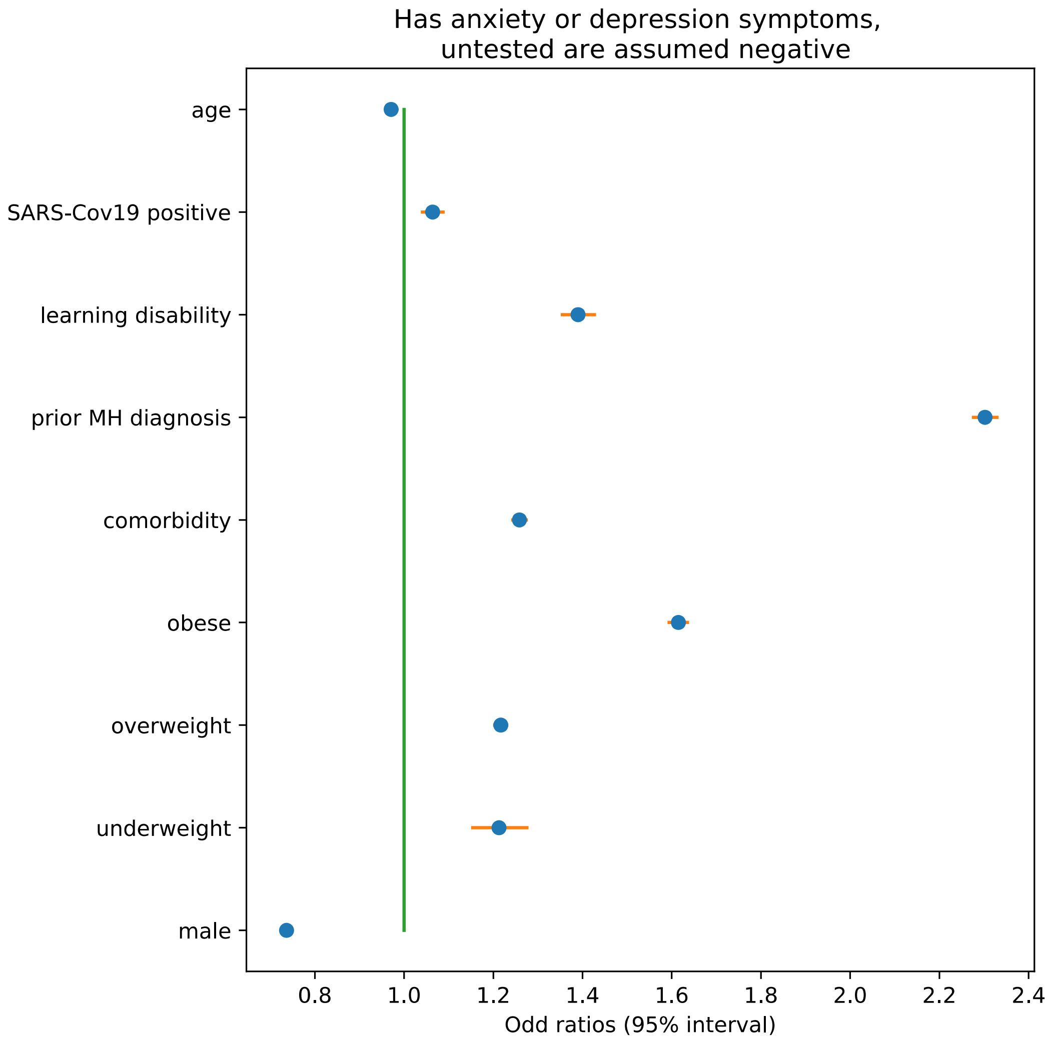

Supplementary Figure 4: Association between age, BMI, male sex, comorbidities, a previous diagnosis of a mental health (MH) condition, learning disabilities, a positive SARS-CoV-2 test result (PCR and lateral flow), and the odds ratio of anxiety/depression symptoms suggested by the results of the mental health survey. Untested cases were assumed negative test results in this sensitivity analysis.
